# Endovascular treatment of low NIHSS score(<6) combined with large vessel occlusion: a meta-analysis

**DOI:** 10.3389/fneur.2026.1730841

**Published:** 2026-06-19

**Authors:** Shiqi Xie, Changli Lou, ZhenHua Liao, Huiqiang Luo, Hai Hu

**Affiliations:** Department of Neurosurgery, Xingguo Hospital, Gannan Medical University, Xingguo, Jiangxi, China

**Keywords:** endovascular treatment, large vessel occlusion, meta-analysis, National Institute of Health Stroke Scale, stroke

## Abstract

**Background and purpose:**

At present, there is still debate about the treatment strategies for acute ischemic stroke patients with low National Institute of Health Stroke Scale (NIHSS) scores (<6 points) and large vessel occlusion (LVO). Our objective is to assess whether endovascular treatment (EVT) could be beneficial in acute ischemic stroke patients with low NHISS scores and LVO.

**Methods:**

We searched the PubMed, Embase, Cochrane Library, and Web of Science databases to obtain articles related to EVT for patients with low NHISS score with LVO until 1 January 2024. The primary outcome was a good functional outcome (modified Rankin Scale [mRS] 0–2). Effect sizes were computed as risk ratio (RR) with random-effects or fixed-effects models. The quality of articles was evaluated through the Cochrane risk assessment tool and the Newcastle–Ottawa Scale.

**Results:**

A total of 2,275 articles were obtained through the search, and articles that did not meet the inclusion criteria were excluded after review of the title, abstract, and full text. Finally, 2 randomized controlled trials (RCTs) and 22 cohort studies met the inclusion criteria. In the EVT group, 77.8% of patients achieved a good functional outcome, while 77.1% of patients achieved functional independence in the best medical treatment (BMT) group. EVT was not associated with excellent functional outcome (mRS 0–1; risk ratio 1.04 [95% CI, 0.97–1.11]) or with a good functional outcome (mRS 0–2; risk ratio 1.00 [95% CI, 0.95–1.04]). Symptomatic intracranial hemorrhage was more common in patients receiving EVT (risk ratio 2.82 [95% CI, 2.18–3.65]). There was no correlation between EVT and 3-month mortality (risk ratio 1.15 [95% CI, 0.93–1.43]).

**Conclusion:**

This meta-analysis shows that, in patients with low NIHSS score combined with LVO, EVT did not demonstrate clear improvement in neurologic outcomes but was associated with an increased incidence of symptomatic intracranial hemorrhage (sICH) compared to BMT.

## Introduction

Stroke is the leading cause of disability and death worldwide after ischemic heart disease (1). Large vessel occlusion (LVO) is a common cause of acute ischemic stroke (AIS). Randomized controlled studies have demonstrated that endovascular therapy (EVT) reduces mortality and disability in patients with AIS due to LVO (2–6). However, current guidelines only recommend EVT to patients with a score of 6 or higher in the National Institutes of Health Stroke Scale (NIHSS) (7), and endovascular treatment is still uncertain to be beneficial in patients with LVO with an NIHSS score of <6 (8). Current American Heart Association/American Stroke Association (AHA/ASA) guidelines consider intravenous thrombolysis (IVT) therapy a reasonable approach when NHISS is <6 (9). However, evidence suggests that, without acute revascularization in these patients, approximately one-fifth of these patients have early neurologic deterioration (≥4 point change in NHISS score), while one-third of them have symptomatic progression (≥1 point change in NHISS score) (10). Even with rescue endovascular therapy (RET), the excellent functional outcome (90-day mRS results (0–1)) will still be lower than that of immediate endovascular therapy (IET) (11).

Several studies have been conducted to evaluate the efficacy and safety of EVT for low NIHSS scores combined with LVO (11–34), but the results remain controversial. For instance, some single-center cohort studies, such as those by Abbas et al. and Haussen et al., have suggested that EVT is safe and effective in this patient subgroup (13, 14). In contrast, other multicenter studies and propensity-matched analyses, such as the study by Goyal et al., have reported no significant difference in outcomes between EVT and BMT (18). Furthermore, Schwarz et al. concluded that EVT might even be associated with worse 3-month functional outcomes compared to BMT in patients with anterior circulation LVO and NIHSS ≤5 (16).

Given the contradictory findings from existing studies and the lack of a comprehensive, updated synthesis of evidence, there is a clear need for a rigorous meta-analysis to clarify the role of EVT in this specific patient population. Therefore, we conducted this meta-analysis with the primary objective to evaluate the efficacy and safety of EVT compared to BMT in patients with low NIHSS scores (≤5) combined with LVO.

## Methods

The study protocol was reported following PRIMA-P guidelines and was registered at PROSPERO (35). The methods and reporting of the systematic review followed PRISMA guidelines. Reporting of statistical data followed SAMPL guidelines (36, 37).

### Data source and search strategy

An electronic search of the EMBASE, Cochrane Library, PubMed, and Web of Science databases was performed to identify relevant studies. We also consulted clinical trial registries (ClinicalTrials.gov). The last search was conducted on 1 January 2024. The primary search terms included “endovascular treatment,” “mechanical thrombectomy,” “stroke,” “cerebrovascular accident,” “NIHSS <6,” “NIHSS ≤5,” and “large vessel occlusion,” among others. For the search strategy, we used a combination of subject terms and free words, which were searched after matching with various databases. Supplementary Table 1 presents the complete search strings used for each database (PubMed, Embase, Cochrane Library, Web of Science) with date limits set from inception to 1 January 2024. In addition, the retrieved references of relevant reviews and meta-analyses were searched again manually.

### Eligibility criteria

Studies that met the following criteria were included: (1) study type: prospective or retrospective studies; (2) study population: age > 18 years; patients with large vessel occlusion induced AIS and NHISS scores ≤5; (3) study outcome: at least one functional outcome at 3 months; (4) study design: patients were assigned to two groups, where one received EVT and the other received best medical treatment. The exclusion criteria were as follows: (1) studies with fewer than five patients in each group; (2) basic research, reviews of animal experiments, META analysis, systematic evaluation of literature and case reports reviews, letters, conference abstracts, and case reports; (3) studies with no access to full text or data extraction; and (4) studies with poor quality or incorrect statistical methods.

### Study selection and data collection

Two researchers (LZH and LCL) independently screened titles and abstracts, extracted data, and assessed study quality using the Newcastle–Ottawa Scale (NOS) for cohort studies and the Cochrane risk-of-bias tool for RCTs. We performed sensitivity analyses based on study quality (e.g., excluding low-quality studies, NOS score ≤3) to assess the robustness of the pooled estimates. This scale can evaluate the quality of case–control studies and cohort studies separately, and contains 8 evaluation criteria with a score of 9. We considered studies with scores of 0–3, 4–6, and 7–9 to be low-, medium-, and high-quality studies, respectively. Final data extraction was performed, and if the data were incomplete or difficult to extract, the corresponding author was contacted via email to obtain complete data or necessary assistance. A third researcher (LHQ) could intervene when literature inclusion or data extraction was controversial. The primary contents of data extraction are first author, publication date, study type, study location, baseline NHISS score, vessel occlusion sites, functional outcome at 3 months, symptomatic ICH, mortality at 3 months, and other related detailed characteristics of the included studies.

### Risk of bias assessment and quality of evidence

The quality of the RCTs and risk of bias were evaluated using the Cochrane risk assessment tool. The Grading of Recommendations Assessment, Development and Evaluation (GRADE) system was used to evaluate the overall quality of evidence. When the number of studies included in the analysis exceeded 10, funnel plots and Egger tests were used to detect publication bias. When the number of studies was less than 10, the Egger test was used for continuous data or high heterogeneity outcomes, and the Harbord test was used for binary data with low heterogeneity.

### Effect measures

The efficacy outcome were defined as follows: (1) excellent functional outcome, defined as a modified Rankin scale [mRS] score of 0–1 and (2) functional independence, defined as a modified Rankin scale [mRS] score of 0–2. The safety outcomes were evaluated as follows: (1) symptomatic ICH and (2) all-cause mortality at 3 months. If no clear data were reported in the article, a description of the relevant outcome was recorded.

### Statistical analysis

Statistical analysis was performed using RevMan5.4 and Stata Software (version 16.0). Forest plots were used to assess the difference between EVT and medication, described by ratio ratios and their 95% confidence intervals. Heterogeneity between studies was assessed by Cochran Q test and I^2^ test, taking the test criterion as *p* = 0.10. If *P* was >0.10 and I^2^ was <50%, multiple studies could be considered homogeneous, and the fixed-effects model was used to calculate the merged statistic; if *P* was ≤0.10 or I^2^ was ≥50%, a random-effects model was applied. To explore potential sources of heterogeneity, we performed meta-regression and leave-one-out sensitivity analyses for outcomes with substantial heterogeneity (I^2^ ≥ 50%).

## Results

### Study characteristics and quality evaluation

**Figure 1:**
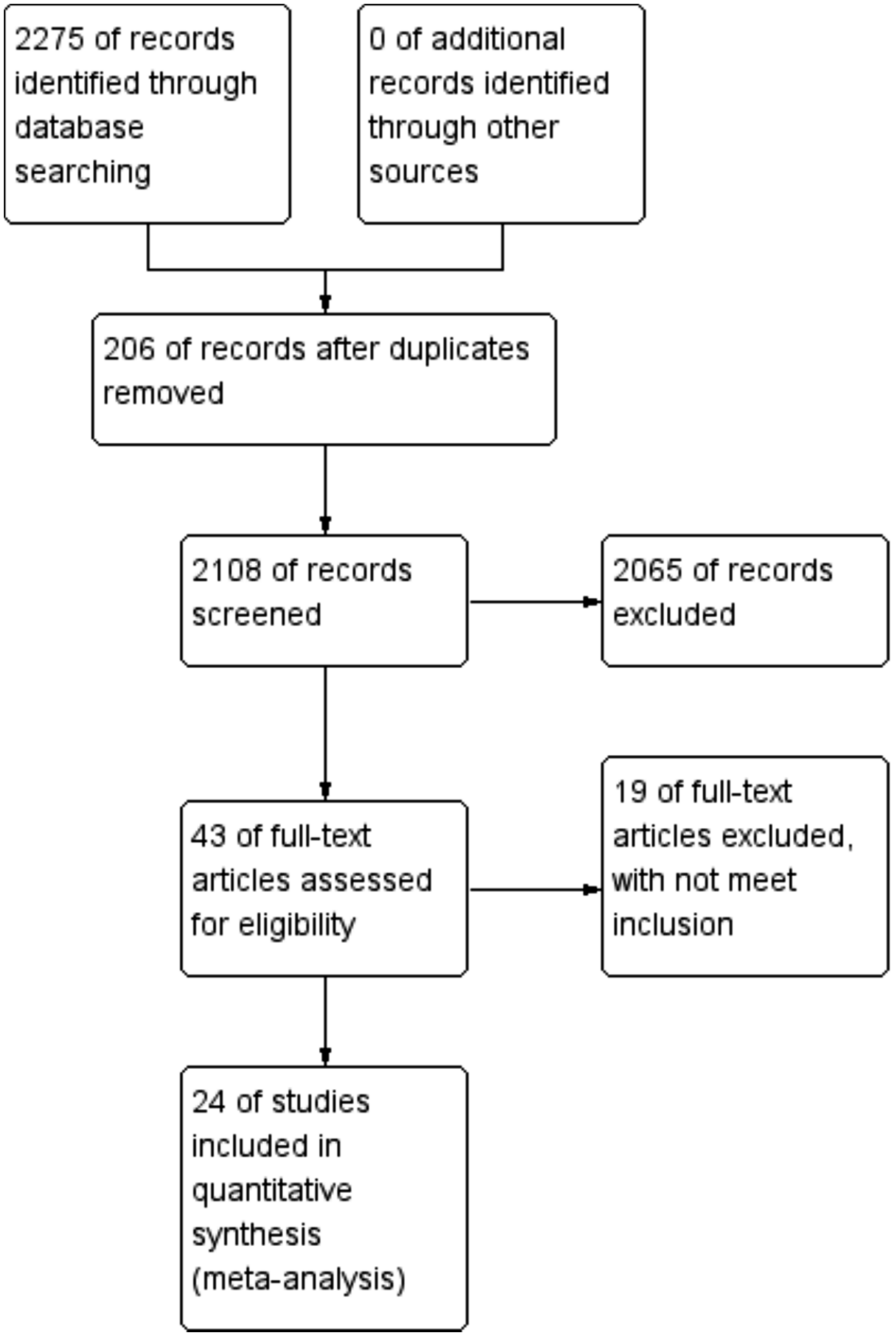
Flowchart presenting the selection of eligible participants.

A total of 2,275 articles were identified from electronic databases and citation searches, of which 167 were excluded as duplicates (Figure 1). Abstracts and titles of the remaining 2,108 articles were manually screened, of which 2,065 studies were excluded due to irrelevance. The full text of 43 articles was retrieved and screened. After reading the full text, 19 articles did not meet the inclusion criteria for exclusion. Finally, 24 articles were eligible, with 3,161 and 4,772 patients treated with EVT and medication, respectively. Basic clinical information such as authors of the included studies, country, year and month of publication, occlusion site, pre-stroke baseline, and number of patients in the vascular treatment group and BMT group is shown in Table 1.

**Table 1:** Primary characteristics of studies included in the systematic review (*n* = 24).

Author	Year	Country	Occlusion site	Pre-stroke baseline	EVT(IVT)	EVT(RET)	BMT(IVT)	BMT(RET)
Abbas R (1)	2022	USA	ICA, A1, M1	0–2	41(14)		42(20)	42(3)
Haussen DC (2)	2016	USA	ACA, ICA, M1, M2, BA	0–2	10(6)		22(2)	22(9)
Nagel S (3)	2018	Germany	ACA, ICA, M1, M2, BA	0–2	80(38)		220(114)	220(25)
Kim BJ (4)	2022	South Korea	ICA, A1, M1, M2	/	149(2)		934(115)	
Schwarz G (5)	2023	Italy	ICA, M1	0	619(439)		418(418)	
Heldner MR (6)	2019	Switzerland	ICA, M1	/	65(0)		120(21)	120(26)
Ros VD (7)	2018	France	ICA, A1, M1	0	32(19)	32(1)	24(24)	
Goyal N (8)	2019	Greece	ICA, M1, M2	0–1	113(47)		138	138(74)
Seners P (9)	2020	France	ICA, M1, M2	/	214(214)		384(384)	384(32)
Messer MP (10)	2017	Germany	ICA, M1, M2	/	8(5)		46(40)	46(6)
Manno C^11^	2019	Switzerland	ICA, M1, M2	0–2	137(69)		175	
Alexandre AM (12)	2021	Italy	ICA, M1, M2	0–1	272(109)		41(30)	41(41)
Rui X (13)	2022	China	ICA, M1, M2	0–2	123(67)	123(43)	449(289)	
Sarraj A (14)	2022	USA	ICA, M1, M2	/	247(72)		254(114)	254(27)
Alexandre AM (15)	2022	Italy	M2	0–2	180(54)		208(133)	208(87)
Dobrocky T (16)	2021	Switzerland	M2	/	85(28)		84(84)	
Volny O (17)	2020	Canada	ICA, M1, M2	0–2	139(80)		97(69)	
Wolman DN (18)	2020	USA	ICA, M1, M2	/	17(6)		30(18)	30(3)
Liu FF (19)	2021	China	ACA, ICA, M1, M2, BA	0–2	43(19)		62(26)	
Wang YC (20)	2023	China	ICA, M1	0–2	48(2)	48(48)	55(4)	
Yedavalli VS (21)	2023	Switzerland	ICA, M1, M2	/	11(6)		35(5)	35(2)
BroccolinA (22)	2023	Italy	M2	0–1		87(87)		
Cappellari M (23)	2023	Switzerland	ICA, A1, M1, M2	/	241(241)		441(441)	
Palazzo P (24)	2023	Switzerland	ICA, A1, M1, M2	/	136(136)		397(397)	397(29)

EVT, endovascular treatment; IVT, intravenous thrombolysis; BMT, best medical treatment; RET, rescue endovascular treatment; A1 indicates anterior cerebral artery; ICA, internal carotid artery; M1, middle cerebral artery.

### Excellent functional outcome at 3 months

**Figure 2:**
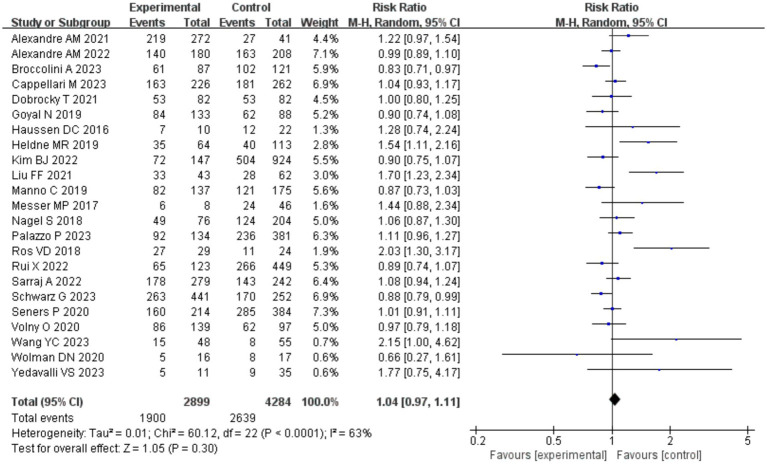
Forest plot presenting the risk ratio of achieving modified Rankin scale (mRS) score of 0–1 at 3 months among patients treated with endovascular treatment (EVT)-treated patients vs. best medical treatment (BMT)-treated patients.

**Figure 3:**
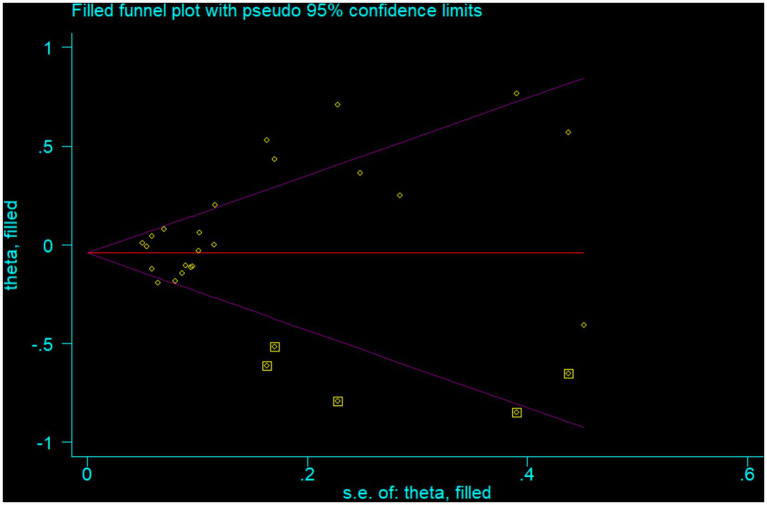
Publication bias graph of achieving modified Rankin scale (mRS) score of 0–1 at 3 months among patients treated with endovascular treatment (EVT)-treated patients vs. best medical treatment (BMT)-treated patients.

A total of 23 studies reported excellent functional outcome after EVT in patients with low NHISS score combined with LVO. The effect sizes were taken as RR values and their 95% confidence intervals; a random effects model was used for its large heterogeneity (I^2^ = 63.0%, *p* = 0.000). The pooled results showed that there was no significant difference in excellent functional outcome between the EVT group and the medication group (RR = 1.04, 95% CI 0.97 to1.11 *p* = 0.30) (Figure 2). Publication bias: The Egger test indicated publication bias (*p* = 0.013 < 0.05); the trim-and-fill method was used to assess the stability of the combined results. Finally, data from five virtual studies were included, and the results show a heterogeneity test: Q = 103.98, *p* = 0.000, (RR = 0.97, 95% CI 0.89 to1.05 *p* = 0.42). Each point in the funnel plot represents a separate study, and the squares represent virtual studies. Results were not statistically significant, so the results were stable (Figure 3).

### Good functional outcome at 3 months

**Figure 4:**
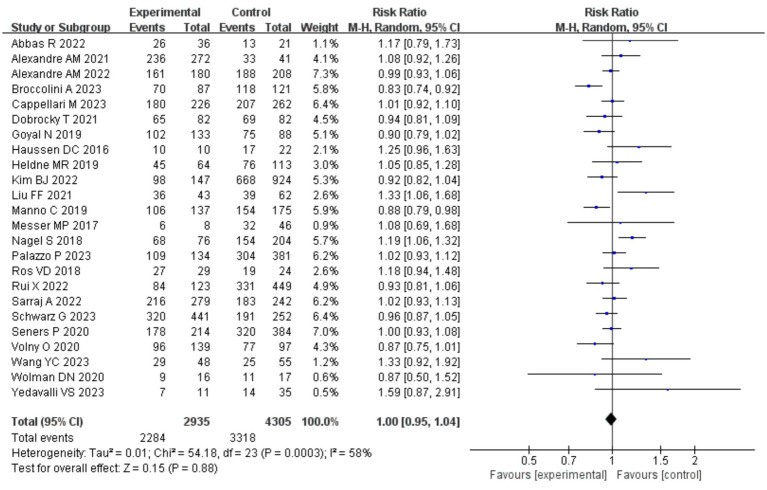
Forest plot presenting the risk ratio of achieving modified Rankin Scale (mRS) score of 0–2 at 3 months among patients treated with endovascular treatment (EVT)-treated patients vs. best medical treatment (BMT)-treated patients.

**Figure 5:**
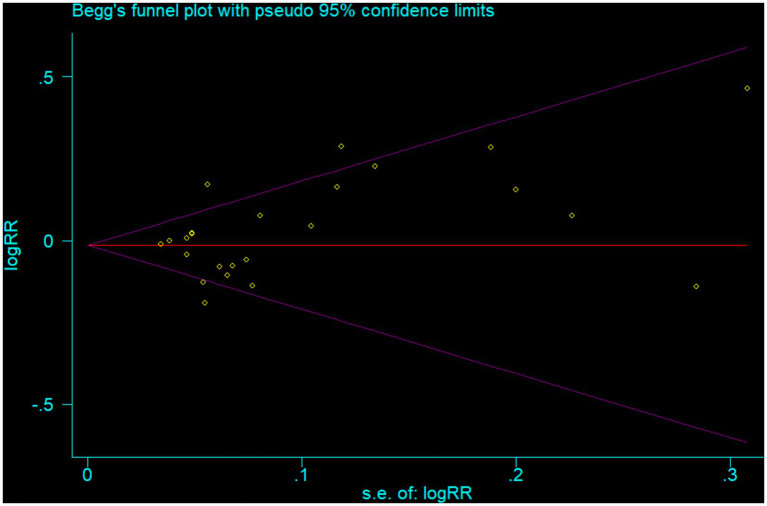
Publication bias graph of achieving modified Rankin Scale (mRS) score of 0–2 at 3 months among patients treated with endovascular treatment (EVT)-treated patients vs. best medical treatment (BMT)-treated patients.

A total of 24 studies reported good functional outcome after EVT in patients with low NHISS score combined with LVO. The pooled results showed that there was no significant difference in good functional outcome between the EVT group and the BMT group (RR = 1.00, 95% CI 0.95 to 1.04, *p* = 0.88). Moderate heterogeneity was present in 23 studies (I^2^ = 57.6%, *p* = 0.000) (Figure 4). Publication bias: The Egger test indicated no significant publication bias (*p* = 0.118>0.05, Figure 5).

### Symptomatic ICH

**Figure 6:**
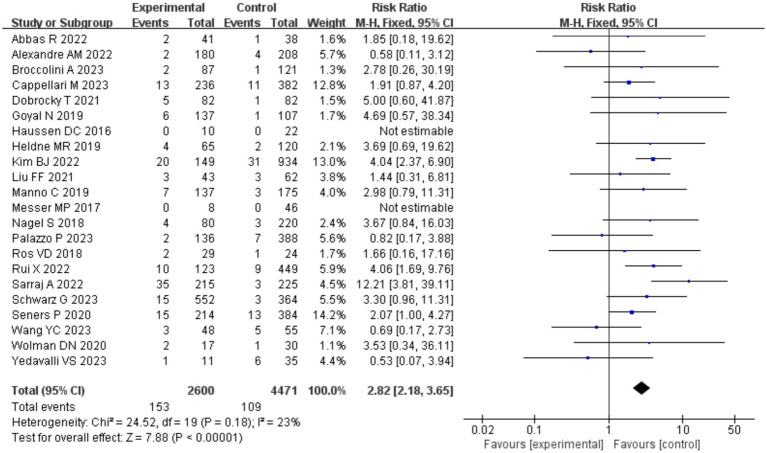
Forest plot presenting the risk ratio of symptomatic cerebral hemorrhage among patients treated with endovascular treatment (EVT)-treated patients vs. best medical treatment (BMT)-treated patients.

Twenty-two studies reported symptomatic ICH, but two of them, the Best Medical Management (BMM) group and the RET group, did not report symptomatic ICH. The pooled results showed that the Endovascular Thrombectomy (EVT) group had a higher risk of symptomatic ICH than the medication group (RR = 2.82, 95% CI 2.18 to 3.65, *p* = 0.000). Mild heterogeneity (I^2^ = 24.5%, *p* = 0.177) was observed in 22 studies (Figure 6).

### Mortality at 3 months

**Figure 7:**
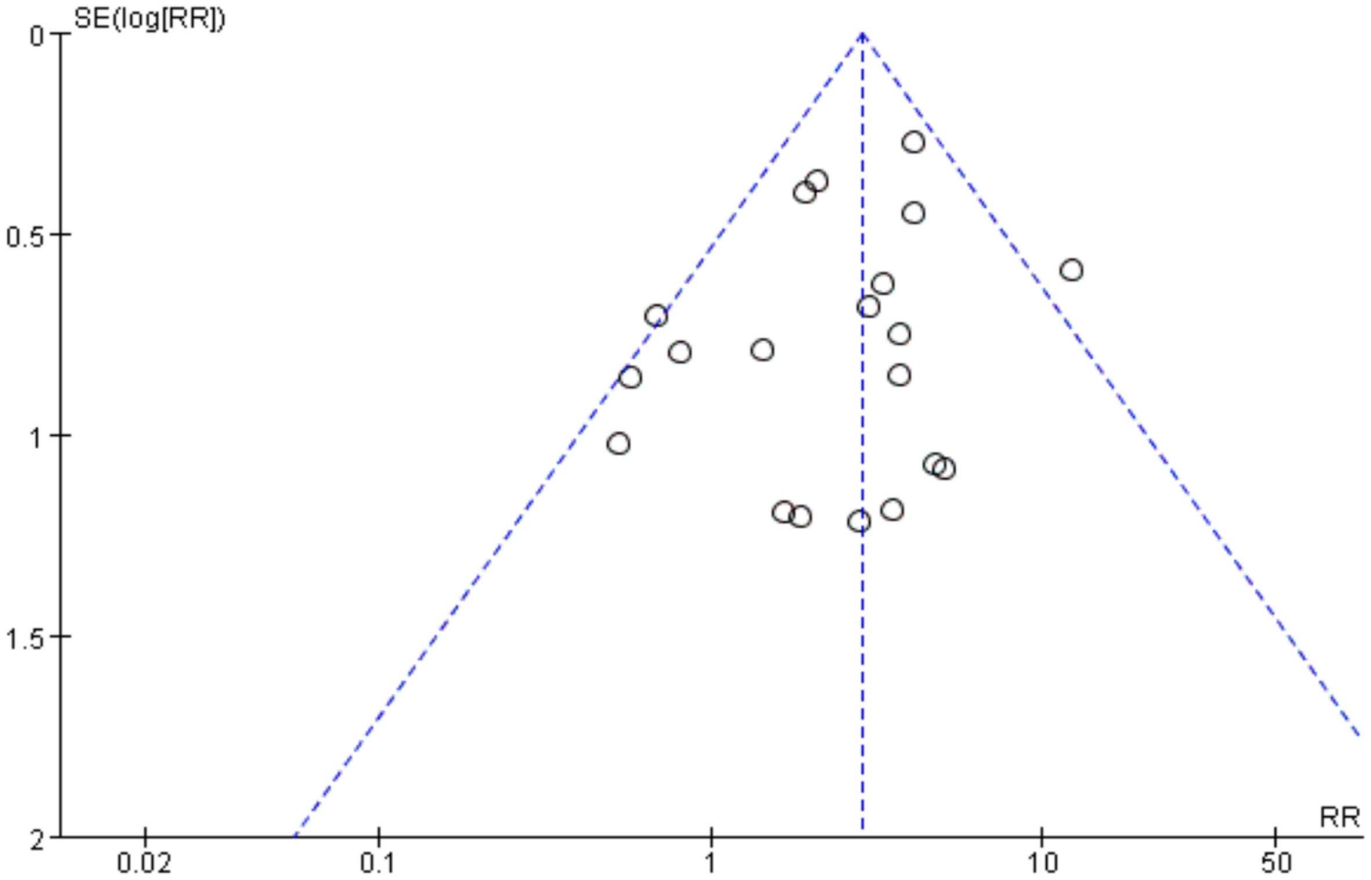
Funnel plot presenting the risk ratio of symptomatic cerebral hemorrhage among patients treated with endovascular treatment (EVT)-treated patients vs. best medical treatment (BMT)-treated patients.

**Figure 8:**
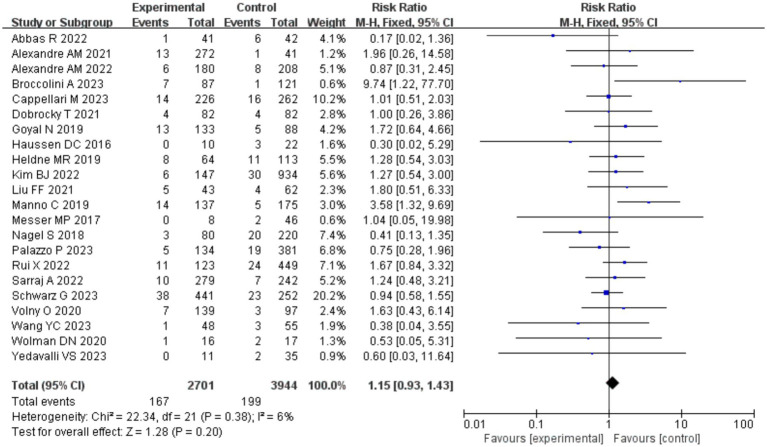
Forest plot presenting the risk ratio of mortality at 3 months among patients treated with endovascular treatment (EVT)-treated patients vs. best medical treatment (BMT)-treated patients.

**Figure 9:**
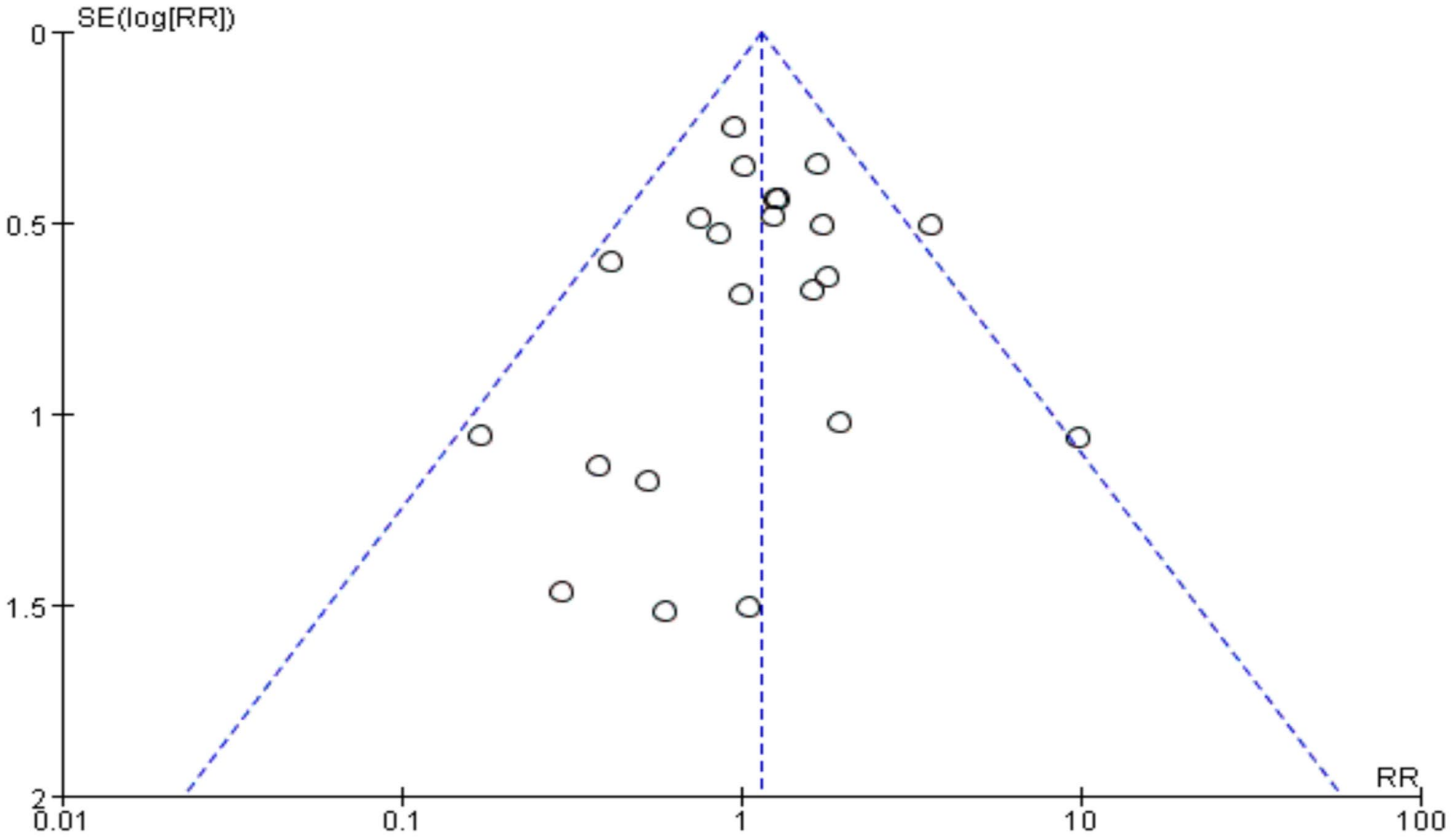
Funnel plot presenting the risk ratio of mortality at 3 months among patients treated with endovascular treatment (EVT)-treated patients vs. best medical treatment (BMT)-treated patients.

A total of 22 studies reported 3-month mortality data. The pooled results showed no significant difference in 3-month mortality between the EVT and medication groups (RR = 1.52, 95% CI 0.97 to 2.39, *p* = 0.07) (Figure 7). Mild heterogeneity (I^2^ = 20%, *p* = 0.24) was observed in 22 studies (Figures 8, 9).

### Subgroup analysis

The 24 studies we included were analyzed in subgroups; the criteria for groups were baseline pre-stroke mRS level, vessel occlusion site, and different treatment designs for rescue endovascular treatment (RET). Results of subgroup analyses are shown: outcomes were similar for different baseline levels of pre-stroke mRS (0–1, 0–2, not described), EVT had no significant benefit on clinical functional outcomes in comparison with BMT in patients with low NHISS scores combined with LVO; however, it also increased the risk of sICH (Table 2).

**Table 2:** Results of subgroup analysis.

Subgroup	Excellent functional outcomes				Good functional outcomes			
	Study, *n*	RR(95% CI)	*p*	I^2^%	Study, *n*	RR(95% CI)	*p*	I^2^%
Pre-stroke baseline								
0–1	5	1.02(0.84, 1.24)	0.825	80.3	5	0.96(0.86, 1.06)	0.419	69.2
0–2	8	1.05(0.91, 1.19)	0.515	64.9	9	1.04(0.94, 1.15)	0.406	74.3
Undescribed	10	1.02(0.92, 1.13)	0.716	61.2	10	1.00(0.96, 1.04)	0.954	0
Vessel occlusion site								
includes M2	19	0.99(0.93, 1.06)	0.753	56.2	19	0.99(0.94, 1.04)	0.608	62.4
Excludes M2	4	1.48(0.90, 2.44)	0.124	88.1	5	1.06(0.94, 1.18)	0.356	31.6
Treatment in the BMT group								
Includes RET	11	1.06(0.96, 1.17)	0.257	60.9	12	1.04(1.00, 1.08)	0.069	19.6
Excludes RET	12	0.99(0.90, 1.10)	0.916	70.9	12	0.95(0.89, 1.01)	0.112	59.5

Subgroup	Symptomatic ICH				Mortality at 3 months			
	Study, *n*	RR(95% CI)	*p*	I^2^%	Study, *n*	RR(95% CI)	*p*	I^2^%
Pre-stroke baseline								
0–1	4	3.20(1.31, 7.81)	0.011	0	4	1.31(0.87, 1.97)	0.197	47.4
0–2	8	2.01(1.24, 3.26)	0.004	25.3	9	1.15(0.80, 1.66)	0.449	46.9
Undescribed	10	3.17(2.29, 4.38)	0	46.8	9	1.05(0.73, 1.49)	0.802	0
Vessel occlusion site								
Includes M2	17	2.99(2.26, 3.95)	0	30.2	18	1.28(0.99, 1.66)	0.056	0
Excludes M2	5	2.04(1.02, 4.07)	0.044	0	4	0.87(0.58, 1.30)	0.495	20.9
Treatment in the BMT group								
Includes RET	11	2.84(1.87, 4.30)	0	49.1	11	0.79(0.54, 1.17)	0.242	0
Excludes RET	11	2.80(2.02, 3.89)	0	0	11	1.39(1.07, 1.80)	0.014	18

RR, risk ratio; CI, confidence interval; RET, rescue endovascular treatment.

Subgroup analyses were performed according to the vessel occlusion site. Five studies included patients with M2, while the remaining 19 studies did not. Compared to BMT, EVT did not improve clinical functional outcomes while increasing the risk of sICH (Table 2).

Subgroup analysis according to whether BMT allowed rescue EVT (RET) showed no significant differences between the two groups in excellent functional outcomes, good functional outcomes at 3 months, and sICH. However, in terms of the 3-month mortality rate (RR = 1.39, 95% CI 1.07 to 1.80, *p* = 0.014), an increased risk of death was observed in the EVT group compared to the BMT group that did not allow RET, whereas a similar outcome was not observed in the group that allowed RET (RR = 0.79, 95% CI 0.54 to 1.17, *p* = 0.242) (Table 2). To address potential immortal-time bias arising from crossover patients (BMT → RET) being analyzed in the EVT group, we performed a sensitivity analysis excluding studies that permitted such crossover; the results remained consistent (Supplementary Table 2).

### Other analysis

Analyses comparing RET with BMT and immediately endovascular therapy (IET) were also conducted. The results revealed that IET significantly improved excellent functional outcomes at 3 months (RR = 1.73, 95% CI 1.20to 2.49, *p* = 0.003) compared with RET, and no significant difference was observed in sICH (RR = 1.74, 95% CI 0.56to 5.46, *p* = 0.345), good functional outcome at 3 months (RR = 1.23, 95% CI 0.99to 1.53, *p* = 0.068), and 3-month mortality rate (RR = 0.67, 95% CI 0.28to 1.62, *p* = 0.375). RET improved excellent functional outcomes at 3 months (RR = 1.18, 95% CI 1.00to 1.39, *p* = 0.044) and good functional outcomes at 3 months (RR = 1.15, 95% CI 1.03to 1.28, *p* = 0.017) compared with BMT, and no significant difference was observed in sICH (RR = 0.59, 95% CI 0.25to 1.39, *p* = 0.23) and 3-month mortality rate (RR = 0.51, 95% CI 0.25to 1.03, *p* = 0.07) (Table 3). To address concerns regarding immortal-time bias arising from the inclusion of patients who crossed over from BMT to rescue EVT (RET) within the EVT group in some studies, we performed a sensitivity analysis. The details of how RET was handled in each included study, along with the results of the sensitivity analysis excluding studies with potential for such bias, are summarized in Supplementary Table 2.

**Table 3:** Results of other analysis.

Other analysis	Excellent functional outcomes				Good functional outcomes			
	study, *n*	RR(95% CI)	*p*	I^2^%	study, *n*	RR(95% CI)	*p*	I^2^%
iET vs. RET	3	1.73(1.20, 2.49)	0.003	12.1	3	1.23(0.99, 1.53)	0.068	0
RET vs. BMM	5	1.18(1.00, 1.39)	0.044	45.9	5	1.15(1.03, 1.28)	0.017	50.4

Other analysis	Symptomatic ICH				Mortality at 3 months			
	Study, *n*	RR(95% CI)	*p*	I^2^%	Study, *n*	RR(95% CI)	*p*	I^2^%
IET vs. RET	3	1.74(0.56, 5.46)	0.345	0	3	0.67(0.28, 1.62)	0.375	0
RET vs. BMM	5	0.59(0.25, 1.39)	0.23	48.4	5	0.51(0.25, 1.06)	0.07	43.9

## Discussion

Currently, approximately 10% of patients with AIS with LVO present with mild stroke (38). Research indicates that neurologic deterioration is significantly higher in patients with large vessel occlusion compared with those without arterial occlusion (39). Approximately 20–40% of patients with large vessel occlusion experience neurological deterioration (10, 40). Even after rescue endovascular treatment after symptomatic exacerbation, some patients with minor strokes experienced residual disability (10).

Our meta-analysis included a total of 7,933 patients, of whom 39.8% underwent EVT and 60.2% received BMT. In the EVT group, 65.5% achieved excellent functional outcome (mRS 0–1) and 77.8% achieved good functional outcome (mRS 0–2), while in the BMT group, the corresponding rates were 61.6 and 77.1%, respectively. These differences were not statistically significant. Regarding safety outcomes, sICH occurred in 153 patients (5.9%) in the EVT group and 109 patients (2.44%) in the BMT group, indicating a significantly higher risk in the EVT group. During the 3-month observation period, mortality was 4.6% (167 patients) in the EVT group and 5.0% (199 patients) in the BMT group, with no significant difference between the two groups.

Our pooled results are consistent with those of previous meta-analyses (41–44), though there are notable differences in patient definitions and inclusion criteria. For example, the meta-analysis by Anagnostopoulos et al. defined mild stroke as NIHSS of ≤8 (44), whereas we adhered to the more commonly used cutoff of NIHSS of ≤5. Additionally, the study by Hou et al. included patients with distal vessel occlusions (M3 or beyond) (43), which were not the focus of our analysis. Compared to the meta-analyses by Qin et al. (41) and Safouris et al. (42), a strength of our study is the inclusion of five recently published articles from national and international registries that were not included in their analyses.

Besides, our subgroup and other analyses revealed intriguing results. First, a recent meta-analysis suggests that EVT may be beneficial for proximal internal carotid artery (ICA) or M1 but not for distal M2 occlusions (45). However, our study did not find this difference, and this may be attributed to the fact that only six early studies were included in their meta-analysis, and additionally, their sample size was small. Second, when RET was part of the interventional arm EVT, EVT was associated with higher 3-month mortality compared to BMT. It is possible that these studies included patients who were originally assigned to the BMT group but were subsequently analyzed in the EVT group because they later received RET, forming an important confounding factor that diminished the benefit of EVT treatment. Therefore, we comprehensively analyzed the safety and efficacy of RET in patients with low NHISS score with LVO. Three studies compared RET with IMT and five studies compared RET with BMM. We pooled studies that RET was associated with lower 3-month excellent functional outcomes compared with IMT, but RET was associated with better 3-month excellent and good functional outcomes compared with BMT, while there were no significant differences in safety outcomes between the two studies. This finding suggests that RET appears to be safe and feasible in case of neurological deterioration, and IMT was better than RET.

At present, it is possible for EVT to improve neurologic function transition in some patients with low NHISS scores combined with LVO, but the risk of hemorrhagic transformation and death, as mentioned above, is equally non-negligible. While MT treatment is associated with a very high probability of immediate discharge to home (46), the incidence of sICH is still as high as 4% and in-hospital mortality as high as 5% with ET treatment, even in the absence of IVT. This is more than enough to offset any benefit of MT treatment for the majority of patients with low NHISS scores who recover satisfactorily (47).

Therefore, based on current evidence, EVT does not appear to offer a net clinical benefit in patients with low NIHSS scores combined with LVO, given the increased risk of symptomatic ICH without a corresponding improvement in functional outcomes. Our findings align with recent meta-analyses (2024–2025) that similarly reported no functional benefit and a higher risk of sICH with EVT in this subgroup. However, it is important to acknowledge the limitations of the included observational studies. Ongoing randomized controlled trials are expected to provide more definitive evidence. Until such data are available, treatment decisions should be individualized, carefully weighing the risk of symptomatic ICH against the potential for early neurological deterioration in each patient.

### Limitations

Nevertheless, there are several limitations to this meta-analysis. First, the majority of the included studies were cohort studies; therefore, bias may still exist owing to a number of unmeasured or unavailable confounders. Second, some studies were derived from overlapping registries (e.g., SITS-ISTR, IRETAS, and MR CLEAN Registry). We verified that no individual patient was counted more than once by ensuring that the included studies reported distinct patient cohorts or non-overlapping time periods. Second, EVT treatment and the specific equipment used for medical management may vary by country and region. Third, the proportion of IVT use varied between the EVT and medication groups, which may reduce the validity of the results. Furthermore, definitions of symptomatic intracranial hemorrhage varied across studies (e.g., ECASS II vs. SITS-MOST criteria), which may have influenced the pooled estimate of hemorrhage risk.

## Conclusion

In conclusion, the results suggest that EVT does not provide clear evidence of benefit in excellent functional outcomes, good functional outcomes, or mortality among patients with low NIHSS scores combined with LVO. Our analysis suggests that RET appears to be a safe and feasible option in cases of neurological deterioration. However, when comparing IET with RET, IET was associated with better functional outcomes at 3 months (RR = 1.73, 95% CI 1.20–2.49, *p* = 0.003), indicating that earlier intervention may be superior to delayed rescue therapy.
