# Supplementary material for: Endovascular treatment of low NIHSS score(<6) combined with large vessel occlusion: a meta-analysis
Source: Front Neurol. 2026 Jun 19;17:1730841. doi: 10.3389/fneur.2026.1730841 (PMC13327980; doi:10.3389/fneur.2026.1730841)
Supplement: Supplementary file 1 [file Table_1.doc]

Supplementary Table 1. Search strategies for each database (inception to Jan 1, 2024)

| **Database** | **Search Strategy** |
| --- | --- |
| PubMed | ("Endovascular Procedures"[Mesh] OR "Thrombectomy"[Mesh] OR (endovascular treatment OR mechanical thrombectomy OR EVT)[tiab]) AND ("Stroke"[Mesh] OR "Ischemic Stroke"[Mesh] OR stroke[tiab] OR AIS[tiab]) AND ("NIHSS"[tiab] OR "National Institutes of Health Stroke Scale"[Mesh]) AND (low OR minor OR \<6 OR ≤5)[tiab] AND ("large vessel occlusion"[tiab] OR LVO[tiab]) NOT (animals[mh] NOT humans[mh]) |
| Embase | 'endovascular treatment'/exp OR 'mechanical thrombectomy'/exp OR (endovascular treatment OR mechanical thrombectomy OR EVT):ti,ab AND 'stroke'/exp OR 'ischemic stroke'/exp OR stroke:ti,ab OR AIS:ti,ab AND (NIHSS OR 'national institutes of health stroke scale'/exp):ti,ab AND (low OR minor OR \<6 OR ≤5):ti,ab AND ('large vessel occlusion' OR LVO):ti,ab AND [humans]/lim |
| Cochrane | (MeSH:[Endovascular Procedures] OR MeSH:[Thrombectomy] OR (endovascular treatment OR mechanical thrombectomy OR EVT):ti,ab,kw) AND (MeSH:[Stroke] OR MeSH:[Ischemic Stroke] OR (stroke OR AIS):ti,ab,kw) AND (NIHSS:ti,ab,kw) AND (low OR minor OR \<6 OR ≤5):ti,ab,kw AND ("large vessel occlusion" OR LVO):ti,ab,kw |
| Web of Science | TS=((endovascular treatment OR mechanical thrombectomy OR EVT) AND (stroke OR AIS) AND (NIHSS) AND (low OR minor OR \<6 OR ≤5) AND ("large vessel occlusion" OR LVO)) AND DT=(Article OR Review) |

Supplementary Table 2. Rescue EVT handling and sensitivity analysis for immortal-time bias

| **Study (Author, Year)** | **RET Allowed in BMT Group** | **Crossover (BMT→RET) Analysis** | **Bias Risk** | **Sensitivity Analysis Results (Excluding High-Bias Studies)** |  |  |  |
| --- | --- | --- | --- | --- | --- | --- | --- |
|  |  |  |  | Excellent Outcome (mRS 0-1) | Good Outcome (mRS 0-2) | sICH | 3-Month Mortality |
| Abbas R, 2022 | Yes | As-treated (BMT group) | Low |  |  |  |  |
| Haussen DC, 2016 | Yes | Intention-to-treat (BMT group) | Low |  |  |  |  |
| Nagel S, 2018 | Yes | Intention-to-treat (BMT group) | Low |  |  |  |  |
| Heldner MR, 2019 | Yes | Intention-to-treat (BMT group) | Low |  |  |  |  |
| Da Ros V, 2018 | Yes | As-treated (EVT group) | High | RR=1.01 | RR=0.99 | RR=2.75 | RR=1.08 |
| Seners P, 2020 | Yes | Intention-to-treat (BMT group) | Low | (95% CI 0.94-1.08) | (95% CI 0.94-1.04) | (95% CI 2.08-3.64) | (95% CI 0.86-1.36) |
| Messer MP, 2017 | Yes | Intention-to-treat (BMT group) | Low | P=0.82 | P=0.67 | P<0.001 | P=0.51 |
| Alexandre AM, 2021 | Yes | As-treated (BMT group) | Low |  |  |  |  |
| Xue R, 2022 | Yes | As-treated (EVT group) | High |  |  |  |  |
| Sarraj A, 2022 | Yes | Intention-to-treat (BMT group) | Low |  |  |  |  |
| Wang Y, 2023 | Yes | As-treated (EVT group) | High |  |  |  |  |
| Broccolini A, 2023 | N/A (RET vs. BMT study) | N/A | N/A |  |  |  |  |
| All other studies | No | N/A | Low |  |  |  |  |
